# Does Intergenerational Educational Mobility Shape the Well-Being of Young Europeans? Evidence from the European Social Survey

**DOI:** 10.1007/s11205-017-1753-7

**Published:** 2017-09-15

**Authors:** Bettina Schuck, Nadia Steiber

**Affiliations:** 1 Institute of Political Science, Heidelberg University, Bergheimer Straße 20, 69115 Heidelberg, Germany; 2 Department of Economic Sociology, University of Vienna, Oskar-Morgenstern-Platz 1, 1090 Vienna, Austria; 3 Wittgenstein Centre for Demography and Global Human Capital (IIASA, VID/ÖAW, WU), International Institute for Applied Systems Analysis, Schlossplatz 1, 2361 Laxenburg, Austria

**Keywords:** Intergenerational mobility, Subjective well-being, Life satisfaction, Diagonal mobility models, Young adults, European Social Survey

## Abstract

Using pooled European Social Survey data (Rounds 4–7, 2008–2014), we investigate the relationship between intergenerational educational mobility and subjective well-being (SWB) for young Europeans (N = 16,050 individuals aged 25–34 from 18 countries). Previous research has been struggling with inconclusive results due to the methodological challenge of disentangling the independent (i.e., ‘net’) effect of social mobility *over and above* the effects of social origin and destination. We contribute to this line of research by contrasting mobility effects estimated in a conventional linear regression framework with net mobility effects estimated by (non-linear) diagonal mobility models (DMM). We show how model selection influences estimates of mobility effects and how different specifications lead to radically different findings. Using DMM, we estimate how intergenerational educational mobility affects the SWB of young Europeans, differentiating between downward and upward mobility and different country groups. Our results suggest that status loss/gain across generations affects young adults’ SWB in addition to the level-effect of ending up in a lower/higher status position only in Continental Europe.

**Electronic supplementary material:**

The online version of this article (doi:10.1007/s11205-017-1753-7) contains supplementary material, which is available to authorized users.

## Introduction

Europe’s current young generation is better educated than any that came before. Increasing levels of education with every new cohort are considered a desirable trend given well-established associations of higher education with longer and healthier lives (Baker et al. 2011; Mirowsky and Ross 2003), greater life-time income, a higher likelihood of being employed and having a rewarding job (Powdthavee et al. 2015; Reynolds and Ross 1998). This results in a positive relationship between educational attainment and individual well-being as well as between expanding mass education and average population health (Baker et al. 2011). Yet, from a sociological perspective, upward mobility—i.e., being more highly educated than one’s parents—would not necessarily be expected to enhance well-being. This goes back to the classic study by Pitirim A. Sorokin in the 1920s that emphasised the benefits of intergenerational upward mobility to society (e.g., increased potential for innovation and adaptation) but also pointed out that such developments may involve psychological costs to upwardly mobile individuals who leave the social class they have been socialized into for a new and less familiar one (Sorokin 1927). Sorokin describes social mobility—in either direction—as a disruptive social experience that causes stress and may undermine well-being (dissociative hypothesis).

The counter-hypothesis is based on the view that in more recent historical periods an increasing emphasis is put on children’s attainment with rising educational aspirations and increased pressure to at least maintain or preferably exceed one’s parents’ attainment. Trends of ever increasing average levels of education across generations raise the costs of not being able to match parental attainment. From this perspective, upward mobility would be expected to be associated with psychological benefits rather than costs (Goldthorpe 1980) and downward mobility is predicted to have negative well-being implications because it creates feelings of personal failure (falling-from-grace hypothesis by Newman 1999). When upward mobility becomes the normative expectation rather than the exception, already a lack of mobility may in fact have a negative impact on well-being (frustrated aspirations). These predictions are in line with those of multiple discrepancies theory (MDT) (Michalos 1985) that describe subjective well-being to be a function of perceived discrepancies between people’s aspirations and the expectations they face, on the one hand, and their achievements, on the other.

According to social mobility theory, individuals’ normative beliefs and attitudes, behaviours and well-being outcomes are influenced by both the status of their parents and their own status (Blau 1956)—in the terminology of social mobility theory: by their origin (O) and destination (D) status. On top, there may be ‘net’ effects of mobility that remain after the influence of O and D has been modelled (Sobel 1981). In contrast to the psychological theories of social mobility mentioned above, that predict either negative or positive net effects of social mobility, the acculturation hypothesis holds that mobility per se does not matter *over and above* the influence of individuals’ origin and destination statuses (Blau 1956). From this perspective, social mobility is seen as a process wherein one’s origin status gradually loses influence while one’s destination status gains in salience as a predictor of outcomes. Once this process is accounted for, net mobility effects, *over and above* origin and destination effects, are held to be zero.

The aims of the present study are: to examine the relative importance of young Europeans’ own level of education and their parents’ educational attainment for their subjective well-being (SWB); and to determine if intergenerational mobility in this regard has an independent effect *over and above* the direct impact of own and parental education. Drawing on data from the European Social Survey for 18 countries, we test if upward mobility has negative or positive well-being implications; if downward mobility has negative implications; or if, alternatively, mobility per se does not matter. A core aim of the study is to explore cross-country differences in intergenerational mobility effects. Our measure of intergenerational social mobility focuses on educational attainment as an important marker of social position. Education is a central determinant of occupational class and income (Blau and Duncan 1967; Card 1999) and the ‘main vehicle for intergenerational reproduction and the main avenue for mobility’ (Torche 2015). Moreover, in comparison to income or occupation, education can also be measured for those not currently employed, which represents an important advantage for studying young adults in contemporary Europe.^1^ Our well-being outcome of interest is young adults’ life satisfaction. The focus of the study is on young Europeans aged 25–34, for whom status gain or loss as compared to parental status is a relatively recent life event, and may be expected to show short and medium-term effects on life satisfaction in young adulthood.^2^

## State of Knowledge

### Previous Research Using Different Approaches

The available evidence on the impact of intergenerational social mobility on well-being outcomes such as happiness, life satisfaction, and health is very mixed. Whereas some studies find that upward mobility is associated with beneficial outcomes (e.g., Campos-Matos and Kawachi 2015; Nikolaev and Burns 2014) others suggest that upward mobility has negative implications for the well-being of the socially mobile (e.g., Hadjar and Samuel 2015; Stacey 1967) arguably because mobile individuals find it hard to adapt to a new class position they have not been socialized into (dissociative hypothesis). Some studies present evidence that appears to support the falling-from-grace hypothesis, showing an association of downward mobility with unfavourable well-being outcomes (e.g., Hemmingsson et al. 1999; Nikolaev and Burns 2014). Finally, some studies suggest that social mobility does not matter (e.g., Marshall and Firth 1999). The incoherence in findings—as we aim to show in this article—is the result of the methodological difficulty of simultaneously estimating the effects of social position of origin (O), social position of destination (D), and mobility between the two across generations (M). In a traditional linear regression approach, only two of the three effects can be estimated given that M is linearly dependent on O and D (Sobel 1981). A substantial part of available research nevertheless relies on conventional linear regression techniques, following one of three approaches:

1. estimating mobility effects while controlling for O (but not for D),
2. estimating mobility effects while controlling for D (but not for O), or
3. estimating mobility effects while controlling for O and D.

Any of these three approaches is unsatisfactory. The first approach provides estimates of mobility effects that are confounded by the influence of own status attainment. This approach therefore tends to provide evidence for positive well-being effects of upward mobility and the reverse for downward mobility (e.g., Campos-Matos and Kawachi 2015; Nikolaev and Burns 2014, Table 10, models 1 and 3). In other words, this approach does not test genuine mobility effects, but a compound of destination and mobility effects, indicating that the upwardly mobile benefit from the well-being enhancing resources attached to their own status compared to their non-mobile comparison group. The second approach provides estimates of mobility effects that are confounded by the effect of parental status attainment, thus tending to arrive at estimates that have been claimed to point to negative effects of upward mobility (dissociative effects, see e.g., Hadjar and Samuel 2015). This approach does also not test genuine mobility effects, but a compound of origin and mobility effects. The estimates for what are termed ‘mobility effects’ in this approach merely reflect the fact that compared to their peers at a similar level of attainment (destination status), the upwardly mobile grew up with less resourceful parents (origin status), who were less able to provide well-being enhancing childhood conditions and to financially and instrumentally support their offspring in adolescence or during the transition to adulthood (e.g., with challenges related to education and training and with labour market entry). The third approach is based on overidentified models and is thus not tenable from a methodological point of view (e.g., Dolan and Lordan 2013; Nikolaev and Burns 2014, models 2 and 4).

The alternative method that breaks the linear dependency of social position of origin (O), social position of destination (D), and mobility between the two across generations (M) are *diagonal mobility models* (DMM), proposed by Sobel (1981). DMM include non-linear diagonal reference terms that allow for a simultaneous estimation of O, D, and M effects (Hendrickx et al. 1993). Details about DMM and their advantages over linear regression approaches are presented in Sect. 4. Studies applying this state-of-the-art statistical technique have investigated intergenerational social mobility outcomes such as political preferences and behaviour (Breen 2001; Weakliem 1992), attitudes toward ethnic minorities (Tolsma et al. 2009), and fertility (Sobel 1985). These studies do not tend to find net mobility effects in either direction. This is also the case for studies concerned more specifically with well-being outcomes. Marshall and Firth (1999) find little evidence for net mobility effects on life satisfaction. Houle and Martin (2011) find that men from a US cohort graduating from high school in 1957 benefitted from upward class mobility out of farming in terms of psychological distress levels. However, this effect was restricted to upwardly mobile sons of farmers and did not extend to other types of mobility.

### Previous Research on Cross-National Differences

Only very few studies have investigated cross-country differences in the well-being effects of intergenerational mobility. Monden and de Graaf (2013) presented the first study aimed to explore the country variation in the relative importance of social origin and destination for self-assessed health in adulthood. Applying a simple dichotomy between Western Europe and the post-socialist countries of Central and Eastern Europe (CEE), the study finds the *relative* importance of fathers’ education—compared to one’s own education—to be greater in CEE than in Western Europe, especially among non-mobile and downwardly mobile individuals. The authors explain this result, arguing that in the transition period reliance on parental resources and social networks was of high importance for individual well-being. The study also tested regional differences in net mobility effects comparing Eastern and Western Europe, yet it neither presented hypotheses nor found such differences. Another comparative study, presented by Campos-Matos and Kawachi (2015), showed that upward mobility (controlling for parental, not for own education) is associated with better subjective health in four welfare regime types, while downward mobility has the opposite effect. To the best of our knowledge, the present study is the first to hypothesise about and investigate cross-country differences in ‘net’ well-being effects of social mobility, *over and above* (i.e., after modelling) origin and destination effects.

## Cross-National Differences

### Country Grouping

The central aim of this study is to investigate differences between countries with regard to the relative strength of mobility effects. Due to limited sample sizes for individual countries, and the limited number of countries available for analysis that do not allow for robust multilevel analyses (Bryan and Jenkins 2016; Stegmueller 2013; Maas and Hox 2005),^3^ we apply a stratified approach. That is, we group the 18 countries under investigation into six country groups, based on frequently used typologies of welfare regimes (Esping-Andersen 1990; Fenger 2007; Ferrera 1996), education systems (West and Nikolai 2013; Green et al. 2006), and central indicators of countries’ socio-economic structures.

Widely used typologies of welfare regimes (Esping-Andersen 1990; Fenger 2007; Ferrera 1996) suggest a differentiation into five country groups: the social-democratic Nordic countries (Denmark, Finland, Sweden, Norway) with universal welfare systems that offer strongly redistributive elements; the conservative Continental European countries (Belgium, France, Germany, the Netherlands) with insurance-based systems of welfare provision that are much less distributive; the Anglo-Saxon countries (Great Britain, Ireland) with residual welfare systems that offer means-tested benefits only; the Southern European countries (Portugal, Spain) with dualist welfare systems that only protect insiders (e.g., Chauvel and Schröder 2014) and are strongly reliant on family-based welfare provision; and the Central and Eastern European (CEE) countries (Estonia, Czech Republic, Hungary, Lithuania, Poland, Slovakia) that form a post-socialist cluster of rudimentary welfare provision. CEE is heterogeneous in terms of market-oriented institution building, however, the Baltic States standing out as a group of countries that have most strongly followed a liberal path, whereas the Visegrád Four (Czech Republic, Hungary, Poland, Slovakia) have introduced an embedded neoliberalism that is associated with much lower levels of social inequality (Bohle and Greskovits 2007). The Czech Republic and Slovakia in fact show surprisingly low levels of income inequality that are comparable to levels found in the Nordic countries (OECD 2012). Based on these socio-economic differences, we thus differentiate two CEE country groups, i.e., the Baltic States and the Visegrád Four. The young Eastern Europeans under investigation in this study were born in socialist times, but were growing up during the transition when inequality was growing fast, especially in the Baltic States (Bandelj and Mahutga 2010).

Due to the setup of the welfare system, the Nordic countries stand out as the most equal. Equality of opportunity is also fostered by the comprehensive school system that has eliminated financial barriers and avoids tracking. This has resulted in enhanced social mobility prospects for those with humble family backgrounds (Esping-Andersen 2015). Moreover, individuals enjoy high degrees of de-commodification and defamilisation (i.e., individual welfare is only weakly dependent on employment status and family support). In the Continental and especially the Southern European countries, by contrast, individual welfare is more strongly dependent on family support and employment status. The two country groups can be differentiated by the degree to which their welfare model is reliant on kinship solidarity, the Southern European model being most strongly based on support across generations (Saraceno 2008). Moreover, the two country groups show great differences in the level of income inequality with much higher levels of inequality observed in the South than in Continental Europe. A further classic feature of the conservative regime is that the goal of social policy is status maintenance rather than inequality reduction. The reproduction of status differences is fostered by selective educational institutions and in particular the strong tracking of the education system. The Anglo-Saxon group features more residual welfare systems and comparatively high levels of income inequality. Similar as in the conservative regime, social status (and here in particular class) has been argued to be an important element of people’s identity in the Anglo-Saxon world (Hadjar and Samuel 2015).

In summary, we distinguish the Nordic countries (Denmark, Finland, Sweden, Norway) from a set of Continental European countries (Belgium, France, Germany, the Netherlands), Southern European countries (Portugal, Spain), Anglo-Saxon countries (Great Britain, Ireland), and two groups of Central and Eastern European countries, i.e., the Baltic States (Estonia, Lithuania) and the Visegrád Four (Czech Republic, Hungary, Poland, Slovakia). This country grouping largely aligns with the differentiation of education regimes as put forward by Green et al. (2006) and West and Nikolai (2013). Based on two dimensions of education systems—inequality of opportunity and expenditure—West and Nikolai (2013) contrast the Nordic countries (comprehensive systems, low levels of selection and tracking, high degree of equality of opportunity) with Continental Europe (stratified systems, high levels of selection and tracking, reproduction of social stratification via the education system). The education systems in the Mediterranean countries are in many ways similar to the ones in Continental Europe, but selection takes place later. The postponement of the age at which youth are tracked into different educational routes would be expected to mitigate the effect of social background on educational outcomes, or in other words, increase chances for social mobility (Lavrijsen and Nicaise 2015). The unstratified education systems in English-speaking countries entail more inequality of opportunity than the comprehensive systems found in the Nordic countries. Finally, many Eastern European countries have established education systems that limit social mobility or in other words, that foster the reproduction of status differences across generations (Kogan et al. 2012; Iannelli 2003). Since this study is less concerned with the cross-national variation in mobility rates than the cross-national variation in the consequences of social mobility, the feature of education systems we are most interested in here is inequality in schooling *outcomes* (e.g., share of pupils lacking basic competencies). It has been shown that the education systems in the Nordic countries are not only more successful in offering greater equality of access to schooling but also greater equality of outcomes compared to the other country groups, and especially Continental Europe, where stratified systems with early academic selection result in hugely unequal outcomes (Van de Werfhorst and Mijs 2010). The Mediterranean and the English-speaking systems tend to fall in-between with medium levels of schooling outcome inequality (West and Nikolai 2013). Both inequality of educational opportunity and inequality of schooling outcomes have been shown to affect income inequality (Checchi and Van de Werfhorst 2014), which is one of our key variables hypothesized to drive cross-country differences.

### Hypotheses

First, we expect *education*-*based well*-*being gradients* to vary across contexts. We expect them to be weaker in countries that feature strongly redistributive and de-commodifying welfare states (H1). This is based on the assumption that education-based well-being gradients are smaller in universal welfare states that offer generous assistance and benefits to all and that tend to exert greater efforts to mitigate the implications of social status differences on well-being inequalities. Moreover, we expect education-based well-being gradients to be weaker in more equal societies, based on the assumption that in societies with low levels of income inequality, social status *per se* is less relevant for well-being given comparatively small education-based income differentials (H2). With view to the countries under investigation, we would thus expect the comparatively smallest education-based well-being gradients in the Nordic countries (offering both a universal welfare state and low inequality levels), followed by the Visegrád Four and Continental European countries (less strongly de-commodifying welfare states and greater inequality compared to the Nordic countries), whereas the largest education-based well-being gradients are predicted for the Southern European and Anglo-Saxon countries, and the Baltic States (rudimentary welfare provision and the highest inequality levels, especially in the Baltic States).

Second, we expect the *relative importance of own and parental education* (i.e., the relative weight of origin and destination status) to vary across contexts. Based on the assumption that in lack of state support for securing individual well-being (e.g., in case of illness or unemployment), individuals are more reliant on family resources and support, we would expect parental education to have greater weight relative to own education in weakly de-commodifying welfare states (H3). Moreover, we would expect the weight of parental education to be greater relative to own education in societies that feature greater levels of inequality (H4). This is based on the following assumptions: Childhood circumstances and family support in adolescence are decisive for well-being outcomes in early adulthood. In countries that feature high levels of inequality, parental education is more strongly linked to the level of resources that parents have available for supporting the well-being of their offspring (Monden and de Graaf 2013). With view to the countries under investigation, we thus expect to find a greater relative weight of origin status in Southern Europe, the Anglo-Saxon countries, and the Baltic States, that all feature weakly de-commodifying welfare states and high levels of inequality, whereas in the Nordic countries, we expect origin effects to be least pronounced (Beller and Hout 2006; Esping-Andersen 2015). We would furthermore expect reliance on the family—and thus parental status—to be of greater relative importance for the well-being of young adults where low levels of de-commodification are combined with high levels of unemployment (H5). Origin effects may thus be particularly strong in Southern Europe, given the extremely high levels of unemployment among the young, and also for the fact that the welfare system is strongly based on the principle of subsidiarity and familialism (i.e., strong institutionalized reliance on family support for welfare).

Third, we expect the importance of *net mobility effects*—that remain when accounting for origin and destination effects (Sobel 1985)—to vary across contexts. A positive net effect of upward mobility would suggest that in addition to the positive implications of attaining a high social position (destination effect) the very fact that this attainment supersedes parental attainment has a beneficial psychological effect (fulfilled aspirations). A negative net effect of upward mobility (dissociative effect) would remain once account is taken of potentially positive destination effects. Finally, a negative net effect of downward mobility would suggest that failing to match parents’ level of education is psychologically taxing (falling-from-grace), over and above the negative effect of the lower status attainment. We may expect to find such psychological mobility effects to be most pronounced in societies where social status is an important element of people’s identity (H6). A strong class consciousness has been argued to be a feature of the Anglo-Saxon world (Hadjar and Samuel 2015). And we may also expect social mobility to have comparatively strong psychological effects in the conservative welfare states of Continental Europe, where social policy and educational institutions are geared toward the maintenance of social status differences and where we find limited social mobility prospects for those with humble family backgrounds. A related final hypothesis is that intergenerational mobility is less likely to have psychological implications in more fluid societies that feature higher rates of mobility (H7). Upward social mobility effects are less likely to be felt in contexts where such intergenerational status trajectories are very common (Goldthorpe 1980) and represent the normative expectation. More generally, the higher the prevalence of certain intergenerational social mobility trajectories, the lower their expected well-being implications (see also Newman 1999).

## Data and Methods

### Data and Sample

We draw on pooled data from the European Social Survey (ESS) Rounds 4–7 collected via face-to-face interviews in 2008–2014 (ESS ERIC 2016). The ESS draws on random samples of respondents aged 15 and over from the non-institutionalized population. Given its comparable design in all participating countries, it provides the unique opportunity to study cross-national differences in the well-being correlates of intergenerational mobility. Our sample includes data from 18 European countries (see Sect. 3) and is restricted to respondents aged 25–34, not currently in full-time education (i.e., those likely to have attained their final level of education). The sample comprises 16,050 young Europeans providing information about their own and their parents’ educational attainment.

### Measures

Our central explanatory variables are own educational attainment, parental educational attainment, and intergenerational educational mobility as the comparison between the two (i.e., distinguishing non-mobility, downward and upward mobility). We measure educational attainment using a reduced form of the International Standard Classification of Education (ISCED) which was explicitly designed for cross-country comparative analysis in Europe (ES-ISCED, cf. Schneider 2010). We distinguish three educational levels: below upper secondary education comprising ES-ISCED levels I and II (‘low’), upper secondary and post-secondary, non-tertiary education comprising ES-ISCED levels IIIa, IIIb, and IV (‘medium’), and tertiary education consisting of ES-ISCED levels V1 and V2 (‘high’). Parental attainment was assessed by combining information from the mother and father (*What is the highest level of education your mother/father successfully completed?*). We use the higher one of the two attainment levels. For those with missing information on one parent (8%), we use the information on the other parent. Mobility is captured by a categorical variable distinguishing the non-mobile (i.e., same educational attainment as parents), from the upwardly mobile (more highly educated than parents) and the downwardly mobile (less highly educated than parents).

Our outcome of interest is life satisfaction, which is a core dimension of subjective well-being (SWB). Life satisfaction is typically defined as a *cognitive* account of satisfaction with life as a whole (Diener et al. 1999).^4^ It refers to the perceived fulfilment of expectations and aspirations. According to multiple discrepancies theory (Michalos 1985), reported satisfaction is a function of perceived discrepancies between one’s attainment compared to one’s aspirations and also compared to the achievements or expectations of others. In this study we are particularly interested in the role of education in shaping life satisfaction, i.e., young adults’ educational attainment compared to their reference groups such as their peers and parents. In addition to educational attainment, life satisfaction has been shown to be shaped by other factors such as access to employment, income, socio-economic status, the quality of social relations (e.g., being married, having friends), and subjective health (Böhnke 2008; Diener et al. 1999). These factors are among the core mechanisms through which education shapes people’s satisfaction with life (Powdthavee et al. 2015). Despite its strong cognitive element (i.e., comparison of aspirations with achievements), life satisfaction has also been shown to be an important component of psychological well-being and shows a strong correlation with depression (Headey et al. 1993).^5^ We employ a single-item measure of general *life satisfaction* as our dependent variable, based on participants’ responses to the question: ‘*All things considered, how satisfied are you with your life as a whole nowadays?*’ where 0 means ‘*extremely dissatisfied*’ and 10 *extremely satisfied*. Prior research on the well-being effects of social mobility has commonly used life satisfaction as a measure of SWB (e.g., Hadjar and Samuel 2015) thus allowing for a comparison with previous estimates.

### Analytic Strategy

We compare the results from conventional linear regression models with those from diagonal mobility models. In the aim to test cross-national differences in effects, we take a stratified approach and run all models separately for each of the six country groups.

In a first step, we replicate previous findings from mobility research using a conventional linear regression framework. This includes (1) models estimating ‘mobility effects’ while controlling for parental attainment (origin status) but not for own attainment (destination status), and (2) models estimating ‘mobility effects’ while controlling for own attainment but not for parental attainment. All models control for sex, age in years, country of residence, citizenship *(Are you a citizen of [country]?*), membership in a minority ethnic group (*Do you belong to a minority ethnic group in [country]?*), and ESS round (dummy variables capturing survey waves). Since our interest is in the overall effects of intergenerational mobility, we do not control for potential mechanisms that might mediate the relationship between educational attainment/mobility and subjective well-being, such as employment status or income level.^6^

As outlined above, conventional regression models—and conclusions drawn from their respective results—fail to tackle the challenge posed by the linear dependency of origin, destination and mobility indicators. Omitting one of these three variables of interest leads to uncertainty about what drives the observed effects of the remaining two (i.e., confounding). Results from such standard linear regression models that have been applied in prior studies will serve to show how model selection influences estimates of mobility effects and how different specifications lead to radically different findings.

In a second step, we estimate mobility effects using *diagonal mobility models* (DMM, Sobel 1981), i.e., a specific type of non-linear model that is grounded in sociological theory and allows for a simultaneous modelling of origin, destination and mobility effects (Hendrickx et al. 1993) while breaking their linear dependency. In mobility theory (Blau 1956; Blau and Duncan 1967) it is argued that an individual’s characteristics and behaviours are affected by both origin and destination status. DMM take this as a starting point for model specification. Non-mobile persons, i.e., those located in the diagonal cells of a mobility table, are assumed to build the core of a social position and to best reflect the characteristics of that position (Sorokin 1959). Therefore, the non-mobiles are modelled as the primary reference group for mobile individuals. In technical terms and applied to our research question, DMM model the life satisfaction score $Y_{ijk}$ of respondent k in cell ij in a mobility table of educational origin (i.e., parental education i) by educational destination (i.e., own education j) as a function of the life satisfaction of non-mobile respondents at the level of respondents’ educational origin (cell ii) and of non-mobile respondents at the level of respondents’ educational destination (cell jj). In other words, DMM model respondents’ life satisfaction as the weighted sum of the estimated mean scores in the non-mobile origin group $(\mu_{ii} )$ and the non-mobile destination group $(\mu_{jj} )$. The weights are represented by the non-linear product terms *q* and (*1*–*q*) that denote the relative influence of own and parental education on respondents’ satisfaction $Y_{ijk}$. They are restricted to be non-negative and to sum up to 1. We estimate two nested models. The baseline model (1) does not yet include net mobility effects. The extended model (2) estimates two additional parameters ($\beta_{1}$ and $\beta_{2} )$ for the dummy variables *UP* and *DOWN* that capture net mobility effects and can be interpreted like OLS coefficients.

1 $$Y_{ijk} = q \times \mu_{ii} + \left( {1 - q} \right) \times \mu_{jj } + e_{ijk}$$

2 $$Y_{ijk} = q \times \mu_{ii} + \left( {1 - q} \right) \times \mu_{jj } + \beta_{1} \times UP + \beta_{2} \times DOWN + e_{ijk}$$

The two nested models are estimated for each country group. All models include the same control variables used in the conventional linear regression framework earlier. We use the Akaike Information Criterion (AIC) and a likelihood ratio test (LRT) to assess model fit. The DMM are estimated using the DREF subcommand of the General Nonlinear Models (GNM) package in R (Turner and Firth 2015).

## Results

### Descriptives

Table 1 shows the distributions of our explanatory variables and the outcome of interest for each country group. Similar to what is known from previous studies on broader age groups (Böhnke 2008), average life satisfaction is highest in the Nordic countries and lowest in the Baltic States. In all country groups, intergenerational stability is the most common mobility experience—a fact that is well in line with the assumption of DMM that the group of non-mobiles forms the *core* of a class. Moreover, it underlines the well-established fact that education has a strong tendency to be reproduced across generations (OECD 2014). Upward mobility is most common in Southern Europe (42%) and least common in the Baltic States (25%). Downward mobility, in turn, is most prevalent in the Baltic States (18%) and the Nordic countries (16%). Thus, upward mobility is found to be far more common than downward mobility in all country groups—a finding that corresponds with earlier findings for the broader set of OECD countries (OECD 2014).

**Table 1:** Composition of country groups

Group name	Nordic	Continental	Southern	Anglo-Saxon	Visegrád Four	Baltic States
Countries	DK, FI, SE, NO	BE, FR, DE, NL	ES, PT	GB, IE	CZ, HI, PL, SK	EE, LT
*Life satisfaction score (range 0*–*10), means and standard deviations in parentheses*						
	7.99 (0.03)	7.08 (0.05)	7.09 (0.05)	7.21 (0.08)	7.09 (0.05)	6.59 (0.10)
*Parents’ educational attainment (O), shares in %*						
Low	0.16	0.24	0.69	0.40	0.28	0.10
Medium	0.46	0.57	0.16	0.36	0.56	0.59
High	0.38	0.19	0.15	0.24	0.15	0.31
*Own educational attainment (D), shares in %*						
Low	0.07	0.13	0.39	0.22	0.14	0.12
Medium	0.50	0.61	0.26	0.38	0.54	0.47
High	0.44	0.26	0.35	0.41	0.33	0.41
*Mobility (M)*						
Upward	0.29	0.29	0.42	0.38	0.35	0.25
Downward	0.16	0.13	0.08	0.12	0.07	0.18
Non-mobile	0.54	0.57	0.50	0.50	0.57	0.57
N	2699	4032	2102	1813	3931	1473

*Source*: ESS4-7, weighted results based on own calculations

*O* origin, *D* destination, *M* mobility

### Linear Regression Models

Panel 1 in Table 2 presents the results of conventional linear models regressing life satisfaction on intergenerational educational mobility while controlling for parental education (omitting own education). Concerning origin effects, we find parents’ education to have a significant impact on their offspring’s well-being in all country groups. In line with previous studies (e.g., Campos-Matos and Kawachi 2015), we find positive well-being effects of upward ‘mobility’ and/or the reverse for downward ‘mobility’ in all country groups. Yet, as outlined above, these estimates of ‘mobility effects’ are confounded by the influence of one’s own status attainment on well-being.

**Table 2:** Confounded estimates from linear regression models

	Nordic		Continental		Southern		Anglo-Saxon		Visegrád Four		Baltic States	
	β	SE	β	SE	β	SE	β	SE	β	SE	β	SE
**Panel 1**: ‘Mobility effects’, controlling for parental education (omitting own education)												
*‘Mobility’ (Ref.: non*-*mobile)*												
Upward	0.14	0.08	**0.53**	0.08	**0.45**	0.09	**0.67**	0.11	**0.63**	0.09	**0.86**	0.14
Downward	**−0.21**	0.09	**−0.63**	0.10	**−**0.38	0.20	**−**0.35	0.18	**−0.39**	0.14	**−0.59**	0.15
*Parents’ educational attainment (Ref.: low educated)*												
Medium	0.10	0.09	**0.49**	0.08	0.24	0.13	**0.76**	0.11	**0.53**	0.10	**1.03**	0.20
High	**0.25**	0.12	**1.10**	0.11	**0.63**	0.16	**1.25**	0.16	**1.32**	0.14	**2.04**	0.22
Intercept	**7.23**	0.36	**7.70**	0.35	**6.09**	0.53	**4.80**	0.55	**5.72**	0.41	**4.28**	0.61
ESS round	Yes		Yes		Yes		Yes		Yes		Yes	
Country	Yes		Yes		Yes		Yes		Yes		Yes	
N	2699		4032		2102		1813		3931		1473	
Adj. R-sq	0.02		0.09		0.07		0.07		0.09		0.10	
**Panel 2**: ‘Mobility effects’, controlling for own education (omitting parental education)												
*‘Mobility’ (Ref.: non*-*mobile)*												
Upward	**−**0.01	0.07	**−**0.08	0.07	0.02	0.13	**−**0.10	0.11	**−**0.11	0.09	**−**0.22	0.14
Downward	**−**0.05	0.09	**−**0.04	0.10	**−**0.08	0.18	**0.36**	0.18	**0.30**	0.13	**0.50**	0.17
*Own educational attainment (Ref.: low educated)*												
Medium	0.23	0.13	**0.55**	0.10	**0.37**	0.14	**0.66**	0.14	**0.48**	0.13	**0.98**	0.20
High	**0.35**	0.14	**1.07**	0.11	**0.59**	0.14	**1.33**	0.16	**1.27**	0.15	**2.02**	0.22
Intercept	**7.13**	0.37	**7.68**	0.36	**6.06**	0.53	**4.92**	0.55	**5.78**	0.41	**4.28**	0.61
ESS round	Yes		Yes		Yes		Yes		Yes		Yes	
Country	Yes		Yes		Yes		Yes		Yes		Yes	
N	2699		4032		2102		1813		3931		1473	
Adj. R-sq	0.03		0.09		0.07		0.07		0.09		0.10	

*Source*: ESS4-7, own calculations

In addition to ESS round and country, all models control for age, sex, citizenship, and membership of minority ethnic group. Full tables are available from the authors upon request. Numbers (effects) in bold indicate significant effects (*p* < 0.05). ‘Mobility’ refers to effects of mobility that are confounded by own attainment (panel 1) or parental attainment (panel 2)

Panel 2 presents the results of linear models regressing life satisfaction on mobility while controlling for respondents’ own education (omitting parental education). Concerning destination effects, we find own education to have a significant positive impact on life satisfaction in all country groups. Yet, estimates of ‘mobility effects’ are radically different compared to the first approach (panel 1) with counterintuitive, positive well-being effects of downward ‘mobility’ in the Anglo-Saxon countries, the Visegrád Four, and the Baltic States. This owes to the fact that parental education is not controlled for in these models, rendering strongly biased estimates of ‘mobility effects’.

A third linear regression approach applied in prior research estimates mobility effects while controlling for origin *and* destination status. Since such an approach is not tenable from a methodological point of view (overidentification), we refrain from replicating this type of model. Instead, we turn to non-linear diagonal mobility models (DMM) that provide a solution to the methodological challenge of estimating mobility effects while accounting for both origin and destination effects (Sobel 1981).

### Diagonal Mobility Models

Two nested DMM were fitted for each country group.^7^ Concerning the education-based gradient of well-being in the group of non-mobiles (diagonal effects in Table 3), the baseline models 1 show rising life satisfaction scores with educational attainment. Comparing country groups, the gradient is found to be most pronounced in the Baltic States and least pronounced in the Nordic countries. The weight parameters from models 1 indicate whether young Europeans’ life satisfaction is closer to their non-mobile counterparts in the destination or origin group. Across all country groups, we find respondents’ own education to be more important for their life satisfaction than their parents’ status. The relative weight of parental education is greatest in the Baltic States (q = 0.33), followed by the Visegrád Four (q = 0.26), and the Anglo-Saxon countries (q = 0.25), whereas the influence of parental education is estimated to lie close to zero in the Nordic and the Southern European countries.^8^ Sensitivity analyses suggest that the differences between country groups in education-based well-being gradients and weights shown in the stratified analysis (Table 3) are statistically significant (see Online Appendix 4).

**Table 3:** Estimates from diagonal mobility models

	Nordic				Continental				Southern			
	Model 1		Model 2		Model 1		Model 2		Model 1		Model 2	
	β	SE	β	SE	β	SE	β	SE	β	SE	β	SE
*Weights* ^a^												
O (q)	0.00	0.25	0.00	0.00	0.14	0.08	**0.75**	0.24	0.00	0.00	0.55	0.48
D (1–q)	**1.00**	0.25	**1.00**	0.00	**0.86**	0.08	0.25	0.24	**1.00**	0.00	0.45	0.48
*Net Mobility (Ref.: non*-*mobile)*												
Upward			−0.01	0.07			**0.37**	0.17			0.26	0.23
Downward			−0.05	0.09			−**0.48**	0.17			−0.23	0.24
*Diagonal* ^b^ *(Ref.: low educated)*												
Medium	0.24	0.14	0.23	0.13	**0.58**	0.11	**0.50**	0.10	**0.38**	0.10	0.28	0.19
High	**0.38**	0.14	**0.35**	0.14	**1.13**	0.11	**1.11**	0.11	**0.61**	0.11	**0.63**	0.15
Intercept	**7.57**	0.38	**7.60**	0.38	**7.00**	0.36	**7.07**	0.36	**7.39**	0.52	**7.41**	0.52
AIC	9912		9913		16,550		16,547		8833		8838	
Pr(>Chi)^c^	0.57				**0.03**				0.87			
N	2699				4032				2102			

	Anglo-Saxon				Visegrád Four				Baltic States			
	Model 1		Model 2		Model 1		Model 2		Model 1		Model 2	
	β	SE	β	SE	β	SE	β	SE	β	SE	β	SE
*Weights* ^a^												
O (q)	**0.25**	0.09	0.42	0.26	**0.26**	0.09	0.28	0.25	**0.33**	0.08	**0.59**	0.29
D (1–q)	**0.75**	0.09	**0.58**	0.26	**0.74**	0.09	**0.72**	0.25	**0.67**	0.08	0.41	0.29
*Net Mobility (Ref.: non*-*mobile)*												
Upward			0.23	0.23			0.08	0.20			0.43	0.34
Downward			0.04	0.26			0.09	0.22			−0.14	0.37
*Diagonal* ^b^ *(Ref.: low educated)*												
Medium	**0.74**	0.15	**0.77**	0.15	**0.41**	0.14	**0.43**	0.14	**1.01**	0.23	**1.11**	0.24
High	**1.39**	0.16	**1.38**	0.16	**1.28**	0.15	**1.28**	0.15	**2.06**	0.23	**2.12**	0.24
Intercept	**5.57**	0.54	**5.47**	0.54	**6.02**	0.42	**5.98**	0.42	**4.78**	0.61	**4.63**	0.62
AIC	7653		7655		16,941		16,944		6241		6243	
Pr(>Chi)^c^	0.43				0.57				0.27			
N	1813				3931				1473			

*Source*: ESS4-7, own calculations

All models control for age, sex, country, citizenship, membership of minority ethnic group, and ESS round

Numbers (effects) in bold indicate significant effects (*p* < 0.05)

^a^O pertains to parental educational attainment; D to own educational attainment

^b^Educational gradient estimated for non-mobile individuals; effects for reference group (low educated) are fixed at zero

^c^ *P* value of likelihood ratio test comparing model 2 and model 1

Model 2 tests for the net effects of intergenerational mobility *over and above* the effects of educational origin and destination, finally allowing mobility effects to be separated from mere level effects. For all but the Continental European countries, model fit statistics indicate that adding mobility indicators to the baseline model (comparing model 2 with model 1) does not provide a significantly better fit to the data. Accordingly, upward mobility and downward mobility effects are shown to be non-significant—net of parental and own educational level, and controls. The exception is Continental Europe, where we find significant net mobility effects: downward mobility reduces life satisfaction whereas upward mobility increases it.

The vast majority of intergenerational mobility trajectories happen between adjacent educational categories (see Online Appendix 5). Differences in effects between shorter and longer range mobility trajectories (e.g., mobility from high to medium versus mobility from high to low levels of education) can thus—although theoretically interesting—not be investigated further. The mobility trajectories we investigate are those prevalent in Europe. Finally, sensitivity analyses show that the findings of DMM are invariant to the use of a four-category educational attainment variable used in prior research (e.g., Monden and de Graaf 2013, see Online Appendix 6).

## Discussion and conclusion

Using diagonal mobility models (DMM), this study aimed to disentangle the impact of intergenerational educational mobility on the subjective well-being (SWB) of young Europeans from the effects of their origin and destination statuses (i.e., parental and own level of educational attainment). The prevalence of such intergenerational mobility was found to vary cross-nationally. The comparatively highest rates of upward mobility were found in Southern Europe (42% of young adults aged 25–34) whereas downward mobility was found to be most prevalent in the Baltic States (18%).

SWB was assumed to increase with levels of education all across Europe, but we expected larger education-based well-being gradients in countries featuring weakly de-commodifying welfare states (H1) and high income inequality (H2). In line with such expectations, the smallest gradient was found in the Nordic countries that have established universal welfare states featuring low levels of income inequality, whereas the largest gradients were found for the Baltic States that feature minimal welfare support and very high levels of income inequality.

In terms of the relative importance of parental and own education for shaping young Europeans’ SWB, the theoretical expectation was that the relative weight of parental status will be greater in weakly de-commodifying welfare states that render young adults more reliant on family resources for welfare (H3) and in contexts of high income inequality (H4). Our findings show that young adults’ own education is more important for their well-being than their parents’ in all countries. Yet, in line with expectations, parental status was found to be most important in the Baltic States, followed by the Visegrád Four, and the Anglo-Saxon countries, whereas the relative weight of parental status was found to be close to zero in the Nordic countries. Given the high levels of income equality in the Visegrád countries, support for H3 appears stronger than for H4. Yet, H3 is only partially supported given the small relative weight of parental status found not only in the Nordic countries but also in the Southern European countries that feature weakly de-commodifying welfare states. We found no support for H5 that predicted a substantial weight of parental education in unemployment ridden Southern Europe where young adults who are out of jobs are strongly reliant on parental support. The limited influence of parental education in Southern Europe may be due to the fact that, in contrast to the other country groups, the majority of parents in Southern Europe are low-educated. Hence their level of education may be a weak proxy for their economic welfare and resources. Our results show that own education is the dominant factor for the SWB of young adults also in Southern Europe.

Turning to net mobility effects, *over and above* (i.e., accounting for) origin and destination effects, our results suggest that only in one of the six country groups, i.e., Continental Europe, status loss/gain across generations affects young adults’ SWB *in addition to the level*-*effect* of ending up in a lower/higher status position. This is in line with our expectation that psychological mobility effects are more likely to occur in societies where status maintenance is important for people’s identity (H6). Regarding the direction of mobility effects, results do not support Sorokin’s dissociative hypothesis. Instead, we find upward mobility to show positive and downward mobility to show negative well-being effects. For the other country groups, model fit statistics suggested the absence of net mobility effects, over and above origin and destination effects. The non-significance of net mobility effects in the Southern European, the Anglo-Saxon, and the Visegrád countries would be in line with H7 which posits that psychological mobility effects are less likely to occur in contexts where social mobility is very common and may thus be the normative expectation.

Our study has strengths and limitations. Its focus on young individuals represents a strength for methodological and conceptual reasons. Focusing on the young population ensures that parents’ education history is restricted to the not too distant past, which renders intergenerational comparisons of education attainment more meaningful compared to studies of older populations. Moreover, a study of social mobility effects on *life satisfaction* as a SWB-outcome benefits from a focus on the younger population, based on the assumption that effects of educational attainment and mobility on life satisfaction are more immediate than health effects, for example. This assumption is corroborated by a sensitivity analysis that runs the DMM shown in Table 3 based on a sample aged 35–60. No net mobility effects on SWB are found for this older part of the population.^9^ Another strength of the study is the use of state-of-the-art modelling techniques for the study of intergenerational mobility (DMM) that allow isolating the psychological (‘net’) mobility effects from the level effects of own and parental education. Despite its strengths, the study also has some limitations. First, the number of countries available for analysis restricted options for cross-national comparative analysis to a stratified approach that does not allow for a direct test of hypotheses on moderating contextual factors. Our approach made it admittedly rather difficult to formally disentangle the moderating effects of welfare generosity and income inequality since low welfare generosity tends to be paired with social inequality. A test of hypotheses regarding moderating factors at the country-level (such as, e.g., the role of different educational systems) based on data from a larger set of countries and providing larger sample sizes and longer time series is a potential avenue for future research. Second, our results are based on data from a period of economic crisis (2008–2014). In times of slack demand, education may have lower returns because young individuals stay longer in education as a means to avoid unemployment or because they do not find adequate jobs despite high attainment levels. On the other hand, education protects from unemployment also in times of crisis and in some countries education-based differences in unemployment risks have aggravated in the recession. The mediating impact of economic conditions is thus theoretically ambiguous and an empirical question for future research.

Finally, we would like to conclude with a critical note on the accuracy of the notion of *acculturation* in the context of studies on well-being outcomes (as applied e.g., also by Houle and Martin 2011). It may not be entirely accurate to assume acculturation as the underlying process for studying social mobility effects on well-being outcomes such as life satisfaction (compared to outcomes that are more directly ‘cultural’ such as norms, attitudes or habitual behaviours). Well-being in adulthood may in part be shaped by acculturation, e.g., by a process of socialization into typical health behaviours or other well-being relevant behaviours of significant reference groups (such as parents and later on one’s peers at the social position of destination). Yet, in this study, the core mechanisms assumed to underlie effects of origin and destination statuses on well-being outcomes in young adulthood are resource-based. Parental status can be taken as a proxy for socioeconomic conditions in childhood and adolescence that show long-run effects on adult well-being (Ben-Shlomo and Kuh 2002). Such effects (‘long arm of childhood’) have been shown to remain effective across the life course and do not wane in a process of acculturation or adaption. Later on, own attainment generates or fails to generate well-being enhancing resources. Such destination effects on life satisfaction can also be assumed to be fairly immediate (e.g., life satisfaction may already increase while studying for a higher degree and certainly in case of successful labour market entry). In other words, the genesis of destination effects is not assumed to be only or chiefly dependent on a lengthy process of acculturation. In sum, we view well-being in adulthood as the outcome of additive effects of the relative strength of origin and destination effects, i.e., as an outcome that does not depend on the time since origin status has been left (i.e., an acculturation process). Instead, the relative strength of origin and destination effects is conceptualised to depend on contextual conditions (at the country-level). Net mobility effects are conceptualised as those effects that remain once the effects of social position of origin and the social position of destination have been modelled.

## Electronic supplementary material

Below is the link to the electronic supplementary material.

Supplementary material 1 (PDF 766 kb)

## Footnotes

1 Given the large proportions of young Europeans out of employment in the time frame of this study (especially in Southern Europe), the occupational status of a substantial share of our sample cannot be measured. Analyses of intergenerational occupational mobility thus suffer from sample selection bias.

2 In contrast to life satisfaction, physical health would be assumed to be affected only in the longer term.

3 Several simulation studies (Stegmueller 2013; Maas and Hox 2005) have investigated the effect of sample sizes on the accuracy of parameter estimates and respective standard errors. All of these studies conclude that a sample size of 18 countries by far falls below the minimum number of units at level 2 (in our case: countries). If we were only interested in country fixed effects, a minimum number of 25 countries would be tenable for linear models (Bryan and Jenkins 2016). Since we are interested in cross-level interactions, our sample size at the country level does not allow for robust estimation (Stegmueller 2013; Maas and Hox 2005).

4 A different conception of life satisfaction contains both cognitive and affective elements, suggesting that people who are satisfied with their lives ‘feel’ content with their lives in addition to the cognitive appraisal of their lives in terms of goal attainment (Hall 2014). To test if our results are more strongly based on the cognitive or the affective component of life satisfaction, we run a robustness analysis using an indicator of SWB that combines happiness and life satisfaction and that thus has a stronger affective element. The results are very similar in substantive terms compared to the results based on the life satisfaction indicator (see Online Appendix 1).

5 In the sample of analysis of this study, life satisfaction correlates with happiness (11-point scale) and the CESD8 depression scale at 0.71 and −0.43, respectively (analysis based on data from waves 6 and 7 of the ESS).

6 Analyses including potential mediating mechanisms are available in Online Appendix 7.

7 See Online Appendix 2 for results of models based on a pooled sample.

8 The finding that origin status has no impact on life satisfaction in the Nordic and Southern country groups (i.e., origin weights lie on the lower boundary of the theoretically possible interval of 0–1), is corroborated by linear regression models that test for the effect of own and parental education (see Online Appendix 3).

9 Regression tables are available upon request from the authors.
