# Supplementary material for: Does Intergenerational Educational Mobility Shape the Well-Being of Young Europeans? Evidence from the European Social Survey
Source: Soc Indic Res. 2017 Sep 15;139(3):1237–55. doi: 10.1007/s11205-017-1753-7 (PMC6156761; doi:10.1007/s11205-017-1753-7)
Supplement: Supplementary file 1 — Supplementary material 1 (PDF 766 kb) [file 11205_2017_1753_MOESM1_ESM.pdf]

Does Intergenerational Educational Mobility Shape the Well-Being of Young Europeans? Evidence from the European Social Survey

Online Appendix

Appendix 1: Main DMM analyses using a two-item scale of SWB as dependent variable

Table A.1: Estimates from DMM predicting self-reported well-being (outcome: SWB - 2-item-scale [1])

	Nordic				*Cont*				*South*				*Anglo*				*V4*				*Baltic*			
	Model 1		*Model 2*		*Model 1*		*Model 2*		*Model 1*		*Model 2*		*Model 1*		*Model 2*		*Model 1*		*Model 2*		*Model 1*		*Model 2*	
	β	*SE*	*β*	*SE*	*β*	*SE*	*β*	*SE*	*β*	*SE*	*β*	*SE*	*β*	*SE*	*β*	*SE*	*β*	*SE*	*β*	*SE*	*β*	*SE*	*β*	*SE*
Weights [2]																								
O (q)	0.00	0.35	0.00	0.00	0.17	0.09	**0.84**	0.27	0.00	0.00	0.57	0.38	**0.29**	0.09	0.49	0.27	**0.24**	0.10	0.01	0.36	**0.28**	0.08	0.58	0.30
D (1-q)	**1.00**	0.35	**1.00**	0.00	**0.83**	0.09	0.16	0.27	**1.00**	0.00	0.43	0.38	**0.71**	0.09	0.51	0.27	**0.76**	0.10	**0.99**	0.36	**0.72**	0.08	0.42	0.30
Net Mobility *(Ref.: non-mobile)*																								
Upward			0.01	0.06			**0.33**	0.14			0.21	0.11			0.24	0.20			-0.10	0.20			0.42	0.30
Downward			-0.07	0.08			**-0.32**	0.14			-0.20	0.17			0.09	0.23			0.22	0.22			-0.13	0.32
Diagonal [3] *(Ref.: Low educated)*																								
Medium	0.13	0.12	0.10	0.12	**0.42**	0.09	**0.36**	0.08	**0.19**	0.08	0.05	0.14	**0.61**	0.13	**0.64**	0.13	**0.32**	0.12	**0.38**	0.12	**0.77**	0.19	**0.91**	0.21
High	0.23	0.13	0.19	0.12	**0.81**	0.09	**0.81**	0.09	**0.38**	0.09	**0.36**	0.13	**1.15**	0.14	**1.14**	0.14	**0.98**	0.13	**0.99**	0.13	**1.69**	0.20	**1.78**	0.21
Intercept	**7.97**	0.33	**8.03**	0.33	**7.54**	0.30	**7.57**	0.30	**7.87**	0.43	**7.90**	0.43	**6.39**	0.47	**6.28**	0.47	**6.36**	0.36	**6.30**	0.36	**5.28**	0.53	**5.11**	0.54
AIC	9191		9192		14981		14979		7960		7963		7123		7124		15588		15591		5765		5766	
Pr(>Chi)[4]	0.34				0.06				0.54				0.19				0.58				0.22			
N	2,699				4,028				2,098				1,812				3,897				1,466			

Notes: All models further control for age, sex, employment status, country, citizenship, membership of minority ethnic group, and ESS round.
Numbers (effects) in bold indicate significant effects (p<0.05).
[1] The dependent variable SWB is a summative index based on happiness ["*Taking all things together, how happy would you say you are?*"] and life satisfaction ["*All things considered, how satisfied are you with your life as a whole nowadays*"]).
[2] O pertains to parental educational attainment; D to own educational attainment.
[3] Educational gradient estimated for non-mobile individuals; effects for reference group (low educated) are fixed at zero.
[4] P-value of likelihood ratio test comparing Model 2 and Model 1.
Source: ESS4-7, own calculation.

Appendix 2: Main DMM analyses using a pooled sample

In addition to the stratified analyses, we estimated two nested DMM for the pooled sample of all 18 countries (see Table A.2). In line with our findings from the stratified analyses, the pooled model suggests that own education is relatively more important than parental education for young Europeans' life satisfaction. The education-based well-being gradient is substantial, the high educated having on average a 1.07 point higher life satisfaction score than their low educated counterparts. With regard to net mobility effects, model fit statistics indicate a better fit of model 2 as compared to model 1. We find statistically significant positive upward mobility effects and statistically significant downward mobility effects, *over and above* origin and destination effects.

Table A.2: *DMM using pooled data from all 18 countries*

	Model 1		*Model 2*	
	β	*SE*	*β*	*SE*
Weights [1]				
O (q)	**0.17**	0.04	**0.53**	0.13
D (1-q)	**0.83**	0.04	**0.47**	0.13
Net Mobility *(Ref.: non-mobile)*				
Upward			**0.26**	0.09
Downward			**-0.20**	0.09
Diagonal [2] *(Ref.: Low educated)*				
Medium	**0.50**	0.05	**0.48**	0.06
High	**1.07**	0.06	**1.07**	0.06
Intercept	**6.60**	0.19	**6.60**	0.19
AIC	66,530		66,525	
Pr(>Chi) [3]	**0.01**			
N	16,050		16,050	

Notes: controls for age, sex, country, citizenship, membership of minority ethnic group, and ESS round. Numbers (effects) in bold indicate significant effects ($p<0.05$).
[1] O pertains to parental educational attainment; D to own educational attainment.
[2] Educational gradient estimated for non-mobile individuals; effects for reference group (low educated) are fixed at zero.
[3] P-value of likelihood ratio test comparing model 2 and model 1.
Source: ESS4-7, own calculations.

Appendix 3: Effects of own and parental education (using linear regression)

As shown in Table 3 in the main text, DMM estimated origin weights that lie on the boundaries of the theoretically possible interval of 0.00 to 1.00 for the Nordic and Southern European countries. In other words, the estimates indicate that parental education has a zero well-being effect in these two country groups. To test the robustness of this finding, we ran conventional linear models regressing life satisfaction on own and parental education. These models corroborate the finding from DMM of very small and non-significant well-being effects of parental education for both country groups (see Table A.3).

Table A.3: *Effects of own and parental education (using linear regression)*

	Southern		*Nordic*	
	β	*SE*	*β*	*SE*
Own educational attainment *(Ref.: Low)*				
Medium	**0.39**	0.11	0.25	0.13
High	**0.61**	0.12	**0.38**	0.14
Parents' educational attainment *(Ref.: Low)*				
Medium	-0.03	0.13	-0.02	0.09
High	0.00	0.15	-0.03	0.09
Intercept	**6.06**	0.53	**7.12**	0.37
ESS round	yes		yes	
Country	yes		yes	
N	2,102		2,699	
Adj. R-sq	0.07		0.02	

Notes: In addition to ESS round and country, models control for age, sex, citizenship, and membership of minority ethnic group. Numbers (effects) in bold indicate significant effects ($p<0.05$). Source: ESS4-7, own calculations.

Appendix 4: Overview of model fit – interaction effects

For a formal test of significance of differences in effects across country groups, we pooled all data from the 18 countries under investigation and tested interaction effects of our estimates for weights and diagonal effects with the country groups. We compared the fit of a series of nested models using likelihood ratio tests (see Table A.4). Model A, the baseline model, includes only the basic control variables (age, sex, ESS round, citizenship, minority group). Model B, additionally includes dummy variables for the country groups and shows a significantly better model fit. Model C allows control variables to vary across country groups, again significantly improving the model fit. Model D tests for significant differences between origin/destination weights across country groups. The significant improvement in model fit compared to model C suggests that the differences between country groups presented in this study are statistically significant. Model E additionally includes interaction effects between diagonal effects and country groups. The significant increase in model fit suggests significant differences in education-based well-being gradients across country groups. Given that model fit statistics suggest that model 2 shown in Table 3 (i.e., the model including mobility dummies) shows a significantly poorer fit to the data than model 1 in five out of six country groups, we refrain from carrying out a similar test of cross-country differences in net mobility effects.

Table A.4: *Overview of model fit – interaction effects*

Model		*Resid. Df*	*Resid. Dev*	*Df*	*Deviance*	*Pr (>Chi)*
A	Baseline model including only basic controls [1]	16037	63723			
B	A + country group	16032	61407	5	2316.20	0.0000
C	B + interaction basic controls * country group	15993	60988	39	418.50	0.0000
D	C + interaction weights * country group	15989	60949	4	39.07	0.0364
E	D + interaction diagonal effects * country group	15978	60615	11	334.26	0.0000

Source: ESS4-7, own calculations. N=16,050.

[1] Basic controls include age, sex, ESS round, citizenship, and minority group

Notes: Model comparisons are based on likelihood ratio tests.

Appendix 5: Sample composition by country group - detailed

Table A.5: *Sample composition by country group - detailed*

Group name	*Nordic*		*Continental*		*Southern*		*Anglo-Saxon*		*Visegrád 4*		*Baltic States*	
Countries	*DK, FI, SE, NO*		*BE, FR, DE, NL*		*ES, PT*		*GB, IE*		*CZ, HI, PL, SK*		*EE, LT*	
	Mean	n	Mean	n	Mean	n	Mean	n	Mean	n	Mean	n
Parents' educational attainment (O)												
Low	0.16	437	0.24	1,030	0.69	1,448	0.40	723	0.28	1,116	0.10	141
Medium	0.46	1,241	0.57	2,444	0.16	337	0.36	661	0.56	2,215	0.59	874
High	0.38	1,021	0.19	828	0.15	317	0.24	430	0.15	600	0.31	458
Own educational attainment (D)												
Low	0.07	181	0.13	539	0.39	825	0.22	393	0.14	535	0.12	173
Medium	0.50	1,337	0.61	2,626	0.26	545	0.38	684	0.54	2,104	0.47	693
High	0.44	1,181	0.26	1,137	0.35	732	0.41	737	0.33	1,292	0.41	607
Mobility (M)												
Upward	0.29	794	0.29	1,262	0.42	892	0.38	687	0.35	1,391	0.25	370
Downward	0.16	441	0.13	568	0.08	160	0.12	212	0.07	284	0.18	266
Non-mobile	0.54	1,463	0.57	2,473	0.50	1,050	0.50	915	0.57	2,255	0.57	837
Detailed mobility trajectories (M)												
Upward (low -> high)	0.04	95	0.03	134	0.16	331	0.10	188	0.04	174	0.02	34
Upward (low -> medium)	0.09	255	0.14	606	0.18	381	0.13	242	0.14	539	0.05	67
Upward (medium -> high)	0.16	444	0.12	521	0.09	179	0.14	257	0.17	678	0.18	269
Downward (high -> low)	0.01	32	0.01	28	0.01	25	0.02	28	0.00	9	0.01	21
Downward (high -> medium)	0.13	347	0.07	318	0.03	71	0.06	111	0.04	152	0.09	133
Downward (medium -> low)	0.02	63	0.05	221	0.03	65	0.04	73	0.03	123	0.08	112
Non-mobile	0.54	1,463	0.57	2,473	0.50	1,050	0.50	915	0.57	2,255	0.57	837
**N**	2,699		4,032		2,102		1,813		3,931		1,473	

Source: ESS4-7, weighted results based on own calculations. Abbreviations: O-origin, D-destination, M-mobility.

Appendix 6: Main DMM analyses using a 4-category education variable

We conducted sensitivity analyses using a 4-category education variable instead of the 3-category version used in the main analyses (see Table A.6). We distinguish between basic education (less than lower secondary education comprising ES-ISCED level I), lower secondary education (ES-ISCED level II), higher secondary education (ES-ISCED levels IIIa, IIIb, and IV) and tertiary education (ES-ISCED levels V1 and V2). For the Nordic countries, the Visegrád Four, and the Baltic States, cell sizes for the lowest attainment level are, however, too small to allow for meaningful analysis (i.e., the number of respondents with only basic education is smaller than 25 in these country groups). Results for the three remaining country groups are found to be robust against this change in measurement.

Table A.6: *Main DMM analyses using a 4-category education variable*

	Continental				*Southern*				*Anglo-Saxon*			
	Model 1		*Model 2*		*Model 1*		*Model 2*		*Model 1*		*Model 2*	
	β	*SE*	*β*	*SE*	*β*	*SE*	*β*	*SE*	*β*	*SE*	*β*	*SE*
Weights [1]												
O (q)	0.14	0.08	**0.78**	0.20	0.00	0.00	0.00	0.00	**0.25**	0.09	**0.44**	0.19
D (1-q)	**0.86**	0.08	0.22	0.20	**1.00**	0.00	**1.00**	0.00	**0.75**	0.09	**0.56**	0.19
Net Mobility *(Ref.: non-mobile)*												
Upward			**0.43**	0.15			0.03	0.11			0.25	0.18
Downward			**-0.47**	0.15			0.07	0.18			0.03	0.22
Diagonal [2] *(Ref.: Basic education)*												
Lower secondary	0.00	0.23	0.24	0.16	0.05	0.15	0.03	0.17	0.05	0.27	0.10	0.25
Higher secondary	**0.58**	0.21	**0.69**	0.14	**0.42**	0.15	**0.39**	0.17	**0.77**	0.23	**0.90**	0.21
Tertiary	**1.13**	0.21	**1.30**	0.15	**0.64**	0.15	**0.63**	0.17	**1.42**	0.23	**1.50**	0.21
Intercept	**7.00**	0.39	**6.85**	0.37	**7.35**	0.54	**7.34**	0.54	**5.53**	0.57	**5.35**	0.56
AIC	16,552		16,547		8,835		8,839		7,655		7,657	
Pr(>Chi) [3]	**0.01**						0.92		0.35			
N	4,032				2,102				1,813			

Notes: All models control for age, sex, country, citizenship, membership of minority ethnic group, and ESS round.
Numbers (effects) in bold indicate significant effects (p<0.05).
[1] O pertains to parental educational attainment; D to own educational attainment.
[2] Educational gradient estimated for non-mobile individuals; effects for reference group (low educated) are fixed at zero.
[3] P-value of likelihood ratio test comparing model 2 and model 1.
Source: ESS4-7, own calculations.

Appendix 7: DMM analyses including potential mediating mechanisms

Table A.7: *Estimates from DMM including potential mediating mechanisms*

	Nordic				*Continental*				*Southern*				*Anglo-Saxon*				*Visegrád 4*				*Baltic States*			
	Model 1		*Model 2*		*Model 1*		*Model 2*		*Model 1*		*Model 2*		*Model 1*		*Model 2*		*Model 1*		*Model 2*		*Model 1*		*Model 2*	
	β	*SE*	*β*	*SE*	*β*	*SE*	*β*	*SE*	*β*	*SE*	*β*	*SE*	*β*	*SE*	*β*	*SE*	*β*	*SE*	*β*	*SE*	*β*	*SE*	*β*	*SE*
Weights [1]																								
O (q)	0.31	0.61	0.23	0.77	0.42	0.28	**1.00**	0.00	0.00	0.00	0.00	0.00	0.33	0.19	0.93	0.55	0.28	0.15	0.23	0.23	**0.31**	0.11	0.78	0.45
D (1-q)	0.69	0.61	0.77	0.77	**0.58**	0.28	0.00	0.00	**1.00**	0.00	**1.00**	0.00	**0.67**	0.19	0.07	0.55	**0.72**	0.15	**0.77**	0.23	**0.69**	0.11	0.22	0.45
Net Mobility *(Ref.: non-mobile)*																								
Upward			-0.02	0.07			0.11	0.07			0.03	0.13			0.31	0.20			0.05	0.10			0.44	0.34
Downward			-0.04	0.09			**-0.20**	0.09			-0.11	0.17			-0.04	0.23			0.15	0.14			-0.22	0.36
Employment status *(Ref.: employed)*																								
Unemployed	**-0.80**	0.11	**-0.80**	0.11	**-0.99**	0.11	**-0.97**	0.11	**-0.71**	0.12	**-0.71**	0.12	**-0.55**	0.14	**-0.55**	0.14	**-0.43**	0.12	**-0.43**	0.12	-0.38	0.21	-0.39	0.21
Inactive	0.12	0.10	0.12	0.10	-0.06	0.09	-0.05	0.09	0.11	0.19	0.11	0.19	-0.06	0.14	-0.06	0.14	**0.33**	0.09	**0.34**	0.09	0.01	0.14	0.01	0.14
Missing value	**3.69**	1.44	**3.68**	1.44	-0.81	0.61	-0.81	0.61	-1.04	1.34	-1.03	1.34	1.70	1.12	1.61	1.12	0.32	0.44	0.32	0.44	n.a.	n.a.	n.a.	n.a.
Feeling about household's income nowadays *(Ref.: Living comfortably)*																								
Coping	**-0.38**	0.06	**-0.38**	0.06	**-0.69**	0.06	**-0.68**	0.06	**-0.59**	0.11	**-0.59**	0.11	**-0.27**	0.12	**-0.28**	0.12	**-0.57**	0.10	**-0.56**	0.10	**-0.76**	0.14	**-0.76**	0.14
Difficult	**-1.11**	0.11	**-1.11**	0.11	**-1.94**	0.09	**-1.93**	0.09	**-1.27**	0.14	**-1.27**	0.14	**-0.97**	0.15	**-0.99**	0.15	**-1.45**	0.11	**-1.44**	0.11	**-1.68**	0.18	**-1.67**	0.18
Very difficult	**-2.52**	0.21	**-2.52**	0.21	**-2.69**	0.16	**-2.67**	0.16	**-1.70**	0.19	**-1.70**	0.19	**-1.76**	0.19	**-1.77**	0.19	**-2.52**	0.16	**-2.52**	0.16	**-2.44**	0.26	**-2.44**	0.26
Missing value	-0.37	0.59	-0.37	0.59	0.69	0.71	0.69	0.71	-0.83	0.47	-0.84	0.47	-0.93	0.73	-0.93	0.73	**-1.04**	0.42	**-1.06**	0.42	-0.65	0.52	-0.63	0.52
Diagonal [2] *(Ref.: Low educated)*																								
Medium	0.07	0.14	0.06	0.14	0.00	0.11	0.05	0.08	0.14	0.10	0.12	0.14	**0.37**	0.15	**0.40**	0.14	-0.09	0.14	-0.05	0.14	**0.58**	0.22	**0.70**	0.22
High	-0.01	0.14	-0.01	0.14	0.20	0.11	**0.27**	0.10	0.11	0.11	0.08	0.14	**0.63**	0.16	**0.56**	0.17	**0.45**	0.15	**0.45**	0.15	**1.28**	0.23	**1.35**	0.24
Intercept	**8.29**	0.36	**8.31**	0.36	**8.75**	0.33	**8.72**	0.33	**8.48**	0.51	**8.49**	0.51	**6.75**	0.53	**6.67**	0.53	**7.85**	0.41	**7.79**	0.42	**6.52**	0.61	**6.38**	0.62
AIC	9,597		9,601		15,796		15,794		8,647		8,650		7,505		7,506		16,554		16,557		6,111		6,113	
Pr(>Chi)[3]	0.89				**0.03**				0.75				0.25				0.43				0.35			
N	2,699				4,032				2,102				1,813				3,931				1,473			

Table A.7: Estimates from Diagonal Mobility Models including potential mediating mechanisms (continued)

Notes: All models control for age, sex, country, citizenship, membership of minority ethnic group, and ESS round.
Numbers (effects) in bold indicate significant effects (p<0.05).
[1] O pertains to parental educational attainment; D to own educational attainment.
[2] Educational gradient estimated for non-mobile individuals; effects for reference group (low educated) are fixed at zero.
[3] P-value of likelihood ratio test comparing model 2 and model 1.
Source: ESS4-7, own calculations.
